# Porous and Close Packed Supramolecular Assemblies from 2,4-Difluoronitrobenzene with Three Different Linkers and an *n*-Butylamine Cap

**DOI:** 10.3390/ijms241914683

**Published:** 2023-09-28

**Authors:** M. John Plater, Abbie J. Esslemont, William T. A. Harrison

**Affiliations:** Department of Chemistry, University of Aberdeen, Meston Walk, Aberdeen AB24 3UE, UK; a.esslemont.19@abdn.ac.uk (A.J.E.); w.harrison@abdn.ac.uk (W.T.A.H.)

**Keywords:** hydrogen bonding, supramolecular assemblies, crystal engineering, porous

## Abstract

A porous structure formed from sheets with cavities and two close packed structures were crystallised from building blocks prepared from 2,4-difluoronitrobenzene, a diamine linker and *n*-butylamine. The porous structure crystallised from a flexible building block prepared using 1,4-diaminobutane as linker. The close packed structures were prepared using either piperazine or 1,4-bis(aminomethyl)benzene as a linker and have less conformational freedom.

## 1. Introduction

**Figure 1:**
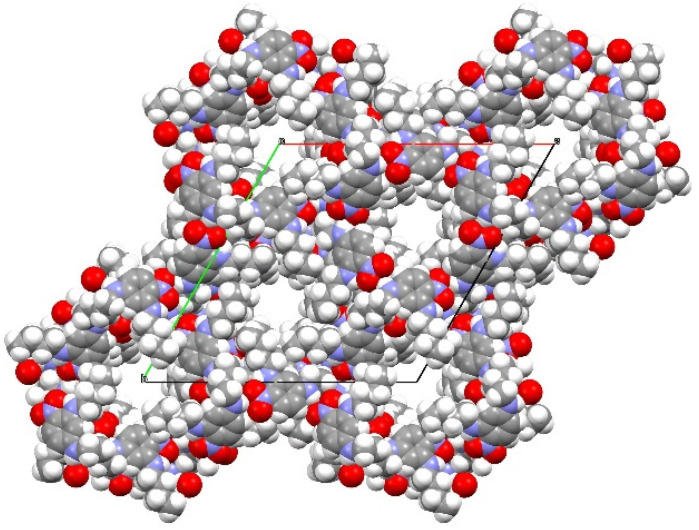

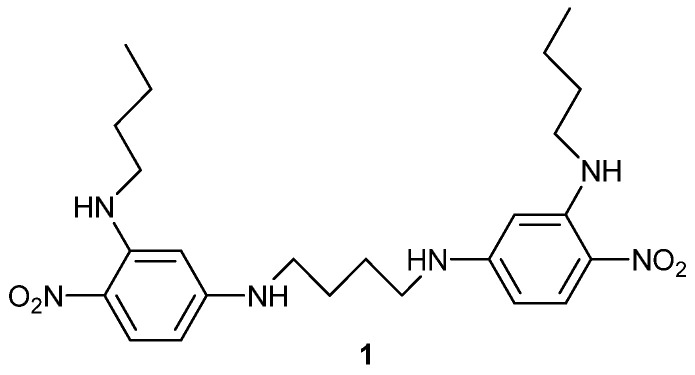
The molecular structure of building block **1** (**bottom**). This was shown by Plater and Harrison by an X-ray single crystal structure determination to be a porous organic ‘zeolitic’ framework (**top**) (Blue sphere is nitrogen, red is oxygen, white is hydrogen and grey is carbon. The red, blue and green lines refer to the a, b and c directions of the unit cell [1].

**Figure 2:**
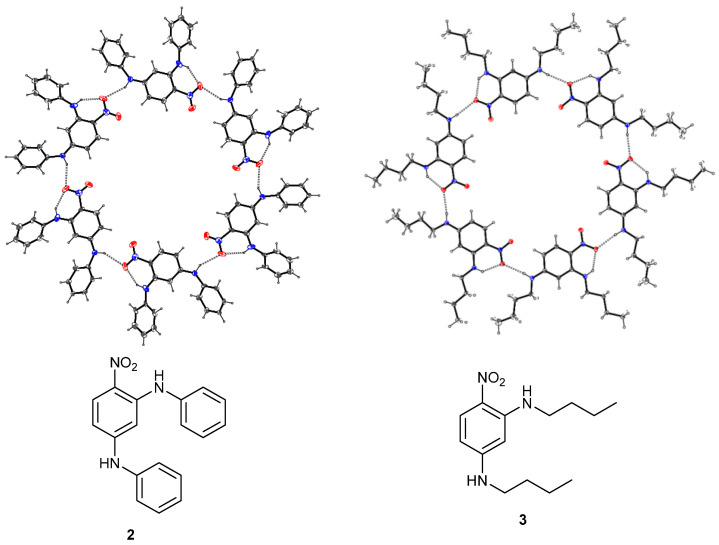
Two examples of hydrogen-bonded hexamers crystallised from 2,4-bis(phenylamino)nitrobenzene **2** and 2,4-bis(butylamino)nitrobenzene **3** (Blue is nitrogen and red is oxygen).

Recently we reported the crystal structures of cyclic hexameric motifs formed from N^1^,N^1^′-(butane-1,4-diyl)bis(*N*^3^-butyl-4-nitrobenzene-1,3-diamine) **1**, 2,4-bis(phenylamino) nitrobenzene **2** and 2,4-bis(butylamino)nitrobenzene **3** in which adjacent molecules are linked by N—H···(O,O) bifurcated hydrogen bonds (Figure 1) [1] or cooperative N—H···O and C—H···O hydrogen bonds (Figure 2) [2,3]. The cyclic hexamers formed from compound **1** and compound **2** are stacked on top of each other so that large one-dimensional channels arise but the cyclic hexamers formed from compound **3** have a staggered stacking arrangement so that channels do not occur in the crystal.

The open frameworks formed from compounds **1** and **2** are examples of porous organic materials comprised of discrete organic molecules between which there are only weak non-covalent interactions. This is quite rare as most organic molecules pack to minimise the void volume [4]. In contrast there are many examples of metal–organic frameworks (MOFs), which have porosity and building blocks which have some rotational flexibility [5,6,7,8,9,10,11,12,13,14].

**Figure 3:**
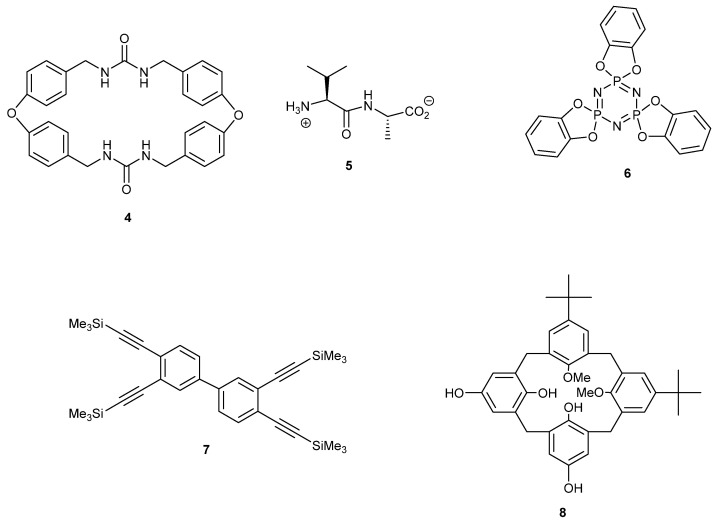
Five examples of ‘organic zeolites’ that crystallise possessing open channels.

Organic compounds that crystallize with channels have been referred to as ‘organic zeolites’ owing to their structural similarity to aluminosilicates [15]. Inorganic zeolites have many applications, such as carbon dioxide capture, hydrogen storage, heterogeneous catalysis and molecular separation [16,17,18,19,20,21]. Soluble precursors required to form organic zeolites may include these applications and extend their range because of solution processing, the choice of components and their functionality (Figure 3) [4]. We and others are also interested in the properties of organic zeolites crystallised from conformationally flexible building block, of which there are many [13]. Cyclic amide host **4** forms cylindrical channels 5–8 Å in diameter stabilised by urea–urea hydrogen bonding and aromatic stacking [22,23]. The material was able to absorb carbon dioxide. Dipeptides, such as compound **5**, crystallise with hydrogen-bonded tubular assemblies forming one-dimensional channels with diameters of 3–5.4 Å. These channels were filled with solvent that could be evacuated by heating [24,25]. This dipeptide has substituents that must play a role in its porosity. Rigid trimer **6** forms crystals with empty 1D channels 5 Å in diameter, which can clathrate solvent and organic guest molecules [26,27]. Tetra (trimethylsilyl ethynyl) biphenyl **7** crystallises, forming narrow channels in three dimensions that interconnect large internal voids of diameter 11 Å [28]. The porosity of the de-solvated crystals was shown by hydrogen and nitrogen absorption. Asymmetric calixarene **8** crystallises in a tubular fashion to form two types of void space filled with water molecules. One void space is a 3D network of channels with diameters of 3.9 and 8.5 Å and the other void space consists of spherical cages of 11.2 Å in diameter connected by narrow channels [29,30]. The host retained its structural integrity upon the removal of the water molecules. Host **6** is rigid and hosts **4–5** and **7–8** are all comparatively rigid compared to the new class introduced here.

## 2. Results

**Figure 4:**
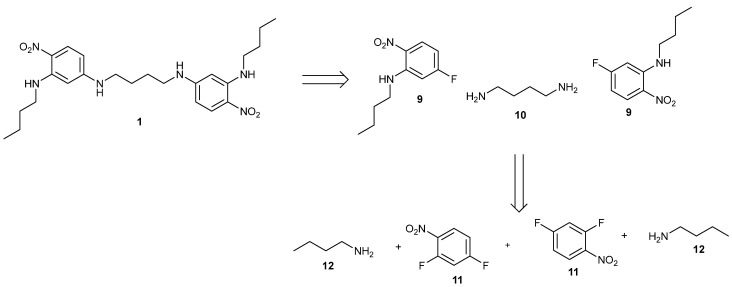
The disconnection of product **1** to its constituent starting materials.

The retrosynthesis of compound **1** is shown in Figure 4. The first disconnection shows that the linker, 1,4-diaminobutane **10**, was added last by displacing the fluorine atoms *para* to the nitro groups. The next disconnection gives the starting materials 2,4-difluoronitrobenzene **11** and butylamine **12**. The first step is faster because of the more reactive and electron-deficient nitro compound **11**. The ortho fluorine group of compound **11** is selectively displaced first before the para fluorine group by butylamine **12**. Presumably the mesomeric and inductive electron withdrawing effect of the nitro group has a greater influence on the ortho positions. This selectivity is key to the success of the synthesis since mixtures are not formed. Also the second fluorine of the mono-substituted product **9** is deactivated by the conjugation of the amino group into the nitro group. However, it is still sufficiently reactive for the second fluorine to be displaced by an amine under reasonable reaction conditions. Ethanol is a good solvent for these displacement reactions, and at 150 °C in a digestion bomb, the second displacement proceeds within 24 h. In this paper, a change of approach has been explored. The diamine linker (**13**, **15** or **10**) is added in first with the more reactive 2,4-difluoronitrobenzene **11,** which should be efficient because an excess of the linker cannot be used. Secondly, an excess of butylamine is coupled with the less reactive para site. The use of butylamine in the last step came about from previous studies, in which we compared methylamine, ethylamine and propylamine [1,2]. The products were much more polar and harder to purify and isolate. Butylamine was the minimum length of chain, which made the products less polar and easy to purify. It is also easily crystallised without disorder, whereas longer chains can be disordered in the X-ray single crystal structure determination. Three linkers were chosen for this study that had different degrees of conformational freedom, which would allow for an exploration of ligand flexibility and porous frameworks. The products were all yellow, with λ_max_ around 400 nm because of the conjugation of the amines to the nitro groups. 

**Figure 5:**
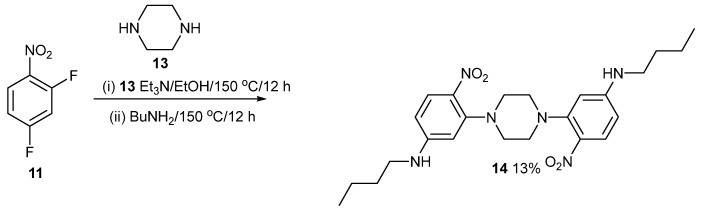
Synthesis of compound **14**.

**Figure 6:**
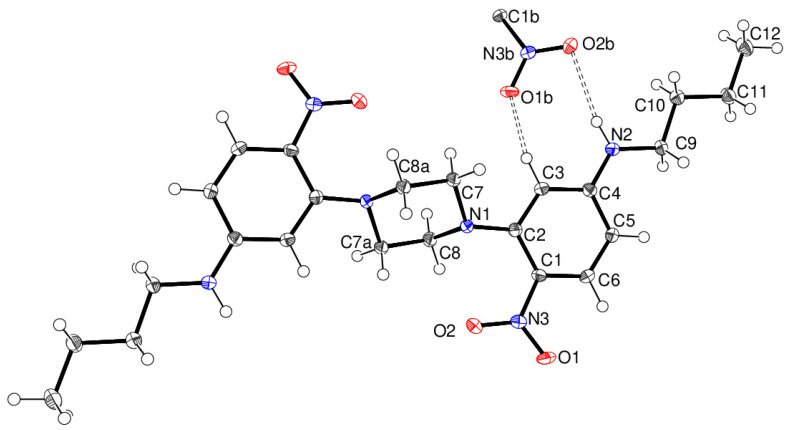
The molecular structure of **14** showing 50% displacement ellipsoids. The cooperative N—H···O and C—H···O hydrogen bonds to the nitro group of an adjacent molecule are shown as double-dashed lines. Symmetry codes: (a) 1–*x*, –*y*, 1–*z*; (b) *x*–½, ½–*y*, ½+*z* (Blue is nitrogen, red is oxygen, white is hydrogen and shaded grey is carbon).

**Figure 7:**
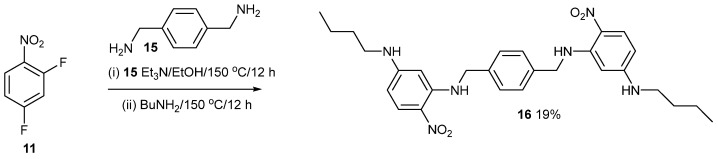
Synthesis of compound **16**.

The ortho fluorine atoms are more reactive than the para fluorine atoms due to nucleophilic displacement. They react this way selectively [1,2,3]. Figure 5 shows the reaction of ½ an equivalent of piperazine **13** with 2,4-difluoronitrobenzene **11** followed by treatment with butylamine **12** to give product **14**. The NMR data are in the Supplementary Materials Figures S1 and S2. Compared to product **1,** the piperazine unit fixes the distance between the aryl rings but still allows for their rotation. The intermediates involved in the first step in Figure 5, Figure 6 and Figure 7 were not isolated because they are characterised indirectly by the second step. Triethylamine is used to mop up acid in the first step and will be present in the second step, but no more was added.

The crystal structure of compound **14** shows that the asymmetric unit consists of half a molecule, with the complete molecule generated by crystallographic inversion symmetry in the space group P2_1_/n. The central piperazine ring adopts its usual chair conformation with the exocyclic N—C bonds in equatorial orientations. The dihedral angle between the piperazine ring (all atoms) and the benzene ring is 49.22 (4)°, and the nitro group is twisted from the plane of the benzene ring by 12.83 (16)°. The *n*-butyl chain adopts an extended conformation. In the extended structure of compound 14, cooperative N2—H1···O2 and C3—H3···O1 hydrogen bonds to different O atoms of the same nitro-group acceptor (Figure 6) generate infinite (101) sheets without identifiable channels or porosity. Figure 7 shows the synthesis of compound **16**. The spacer is longer than piperazine but still has some conformational freedom. 

**Figure 8:**
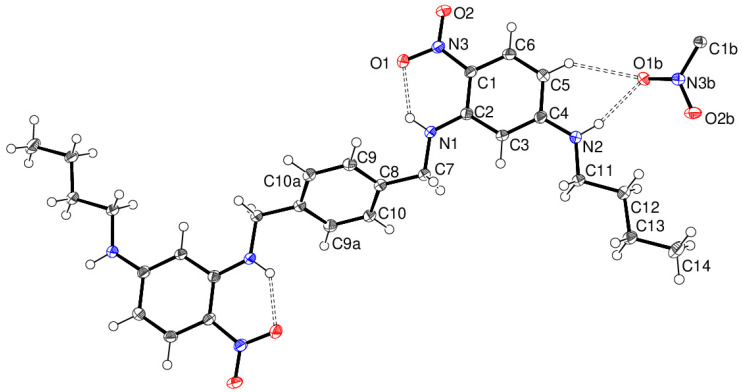
The molecular structure of compound **16** showing 50% displacement ellipsoids. The cooperative N—H···O and C—H···O hydrogen bonds to the same O atom of the nitro group of an adjacent molecule are shown as double-dashed lines. Symmetry codes: (a) 1–*x*, 2–*y*, 1–*z*; (b) ½–*x*, *y*–½, 3/2–*z* (Blue is nitrogen, red is oxygen, white is hydrogen and shaded grey is carbon).

The NMR data for compound 16 are in the Supplementary Materials Figures S3 and S4. The molecular structure of compound **16** consists of a half-molecule in space group *P*2_1_/*n* with the complete molecule generated by crystallographic inversion symmetry (the inversion centre is at 1–*x*, 2–*y*, 1–*z* for the asymmetric molecule). The dihedral angle between the central benzene ring and the pendant nitrobenzene ring is 52.97 (4)°, and the nitro group is close to the plane of its attached ring (dihedral angle = 3.98 (15)°), with this near coplanarity being reinforced by an intramolecular N1—H1n···O1 hydrogen bond. The C2—N1—C7—C8 torsion angle is –178.12 (11)°, and the pendant *n*-butyl chain adopts an extended conformation. In the crystal of compound **16**, cooperative N2—H2n···O1 and C5—H5···O1 hydrogen bonds occur (Figure 8), but unlike compound **14**, atom O1 acts as a ‘double acceptor’ for both hydrogen bonds. This also results in (101) sheets of molecules without any identifiable pores or channels. 

**Figure 9:**
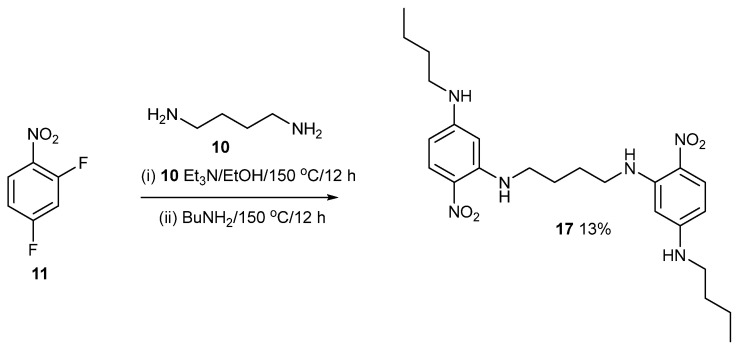
Synthesis of compound **17**.

Figure 9 shows the synthesis of compound **17**. The spacer has the most flexibility in these studies. Compound **17** is an isomer of compound **1,** in which the positions of the bridging and terminal *n*-butyl chains on the nitrobenzene ring are swapped, so here, the two crystal structures can be compared (see below). For compound **1**, butylamine reacted firstly in the ortho positions, followed by the linker in the para position. For compound **17**, the linker reacts firstly in the ortho positions, followed by butylamine in the para positions. Compound **17** is poorly soluble and is unusually polar compared to its isomer **1**. For this reason, only the IR and UV data have been reported, and the crystal structure and it could not be chromatographed with MeOH on silica gel. Compound **1** was purified by chromatography on silica gel with ether/dichloromethane mixture (20:80), which is significantly less polar. The alkylamino-nitro groups in compound **17** must bind to the silica surface or wrap round the silanol groups but they cannot in compound **1**. 

**Figure 10:**
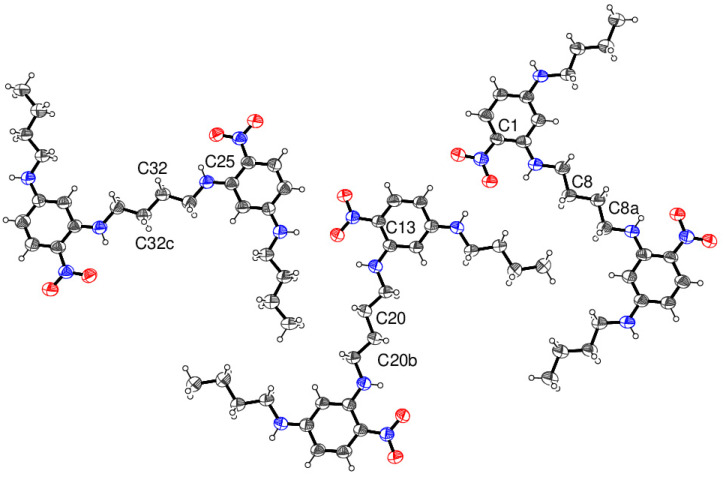
The molecular structure of compound **17** showing 40% displacement ellipsoids with selected atoms labelled. Symmetry codes: (a) 1–*x*, –*y*, –*z*; (b) 2–*x*, 1–*y*, 2–*z*; (c) 2–*x*, 3–*y*, 3–*z* (Blue is nitrogen and red is oxygen).

**Figure 11:**
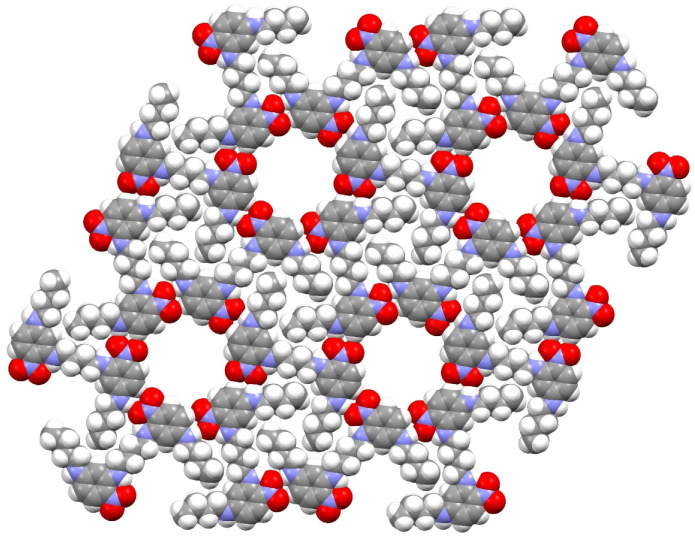
Space-filling representation of four adjacent pores in a (31$\bar{2}$) hydrogen-bonded layer in the structure of compound 17.

Compound **17** crystallises with three half-molecules (containing C1, C13 and C25) in the asymmetric unit, with each molecule completed by crystallographic inversion symmetry (Figure 10); this results in the uncommon situation of *Z* = 3 in space group *P*$\bar{1}$. The central butyl chain adopts an extended conformation in each molecule, as does the pendant *n*-butyl chain. The dihedral angles between the benzene ring and its attached nitro group are 5.2 (3), 0.8 (3) and 0.9 (5)° for the C1, C13 and C25 molecules, respectively; in each case an intramolecular N—H···O hydrogen bond occurs as also seen in **16**. Adjacent molecules in the crystal of compound **17** are linked by cooperative N—H···O and C—H···O hydrogen bonds to different O atoms in an adjacent nitro group in a similar fashion to structure **14**. Unlike compounds **14** and **16**, the extended structure of compound **17** features an infinite network of hydrogen-bonded six-rings of molecules (Figure 11) to generate porous (31$\bar{2}$) sheets with an approximate atom-to-atom pore diameter of 11.4 Å, but from layer-to-layer, groups of three *n*-butyl chains stack on top of a pore, so there is unlikely to be any bulk porosity. A PLATON void-space analysis indicated that 148 Å^3^ per unit cell (7.6% of the total unit-cell volume) could be regarded as free space, which is probably occupied by highly disordered solvent molecules. 

## 3. Discussion

Some key structural features of compounds **1**, **14**, **16** and **17** are summarised in Table 1. 

**Figure 12:**
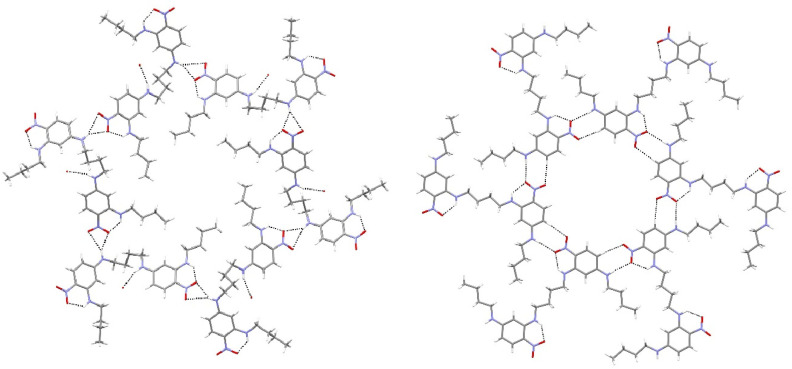
Comparison of (**left**) the hydrophobic channel lined by *n*-butyl groups formed from a hydrogen-bonded hexamer in compound **1** and (**right**) the equivalent hydrophilic channel formed in compound **17** (Blue is nitrogen and red is oxygen).

The key synthon of a group of five atoms consisting of an N—H group (adjacent to the benzene ring) with an ortho C—H group (i.e., H—N—C_ar_—C_ar_—H; ar = aromatic) as cooperative hydrogen-bond donor(s) and a nitro group as acceptor in these related phases shows notable flexibility with bifurcated N—H···(O,O), double acceptor (N—H,C—H)···O and cooperative N—H···O + C—H····O′ (we call these B, D and C modes, respectively; see Table 1 footnote and figures above), which are all possible. However, this *motif* by itself cannot be directly related to the formation of a dense or porous network, as both compound **14** (dense) and **17** (porous) show the same type-C motif. Conversely, both **1** (B mode) and **17** (C mode) show porosity. As noted previously, compounds **1** and **17** are isomers in which the linker and pendant groups have swapped placed on the nitrobenzene ring. The biggest structural difference lies in the conformation of the linking butyl chain, which is very contorted in compound **1** (*a*–*g*–*a*–*g*–*g*) (*a* = anti, *g* = gauche) and centrosymmetric all-anti in compound **17**. A major difference between the extended structures of compounds **1** and **17** arises from the different natures of their pores. In compound **1**, the [001] channels may be assumed to be hydrophobic, being lined by six *n*-butyl chains, but in compound **17**, the (31$\bar{2}$) pores within a single hydrogen-bonded layer must have a more hydrophilic nature, being lined by oxygen atoms (Figure 12). As might be expected, the continuous channels in compound **1** show a far greater bulk porosity (2660 Å^3^ or 19.8% of the total unit-cell volume) than the separated pores in compound **17** (148 Å^3^ or 7.6% of the total unit-cell volume). Other weak intermolecular interactions in these structures should also be briefly considered: in compound **1**, there are no significant aromatic π–π stacking interactions (shortest centroid–centroid separation = 4.3271 (14) Å), and any C—H···π bonds must be extremely weak, with the shortest H···π distance being 2.88 Å. In compound **14**, the shortest π–π centroid separation of 5.0132 (7) Å is far too long to be regarded as a significant interaction, and the shortest C—H···π bond (2.86 Å) is also very weak. Compound **16** shows corresponding values of 4.9853 (8) Å and 2.73 Å for the π–π centroid separation and C—H···π separations, respectively. Finally, compound **17** has a shortest ring-centroid separation of 4.561 (3) Å and a shortest C—H···π contact of 2.69 Å. To this may be added an N—O···π contact with O···π = 3.244 (5), rather less than the van der Waals radius sum of 3.32 Å (assuming a ‘half-thickness’ of an aromatic ring to be 1.6 Å), and N—O···π = 93.5 (3)°, but the significance of such contacts is debated [31]. 

The C—H···O hydrogen bonding observed in compound **17** is unusual because hydrogen is of similar electronegativity to carbon, but it is common in biological systems and has been a topic of discussion [32,33,34,35,36].

## 4. Materials and Methods

IR spectra were recorded on a Thermoscientific Nicolet Summit diamond-attenuated total reflection Everest (ATR) Fourier transform infrared (FTIR) spectrometer (Milton Park, Oxford, UK). Ultraviolet (UV) spectra were recorded using a Perkin Elmer Lambda 25 UV-Vis spectrometer with EtOH as the solvent (Chalfont Road, Buckinghamshire, UK). The term sh means shoulder. ^1^H and ^13^C nuclear magnetic resonance (NMR) spectra were recorded at 400 and 100.5 MHz, respectively, using a Bruker 400 spectrometer (Welland House, Coventry, UK). Chemical shifts, δ, are given in ppm and measured by comparison with the residual solvent. Coupling constants, *J*, are given in Hz. High-resolution mass spectra were obtained at the University of Wales, Swansea, using an Atmospheric Solids Analysis Probe (ASAP) (positive mode) instrument, namely Xevo G2-S ASAP (Waters^TM^, Wilmslow, UK). Melting points were determined on a Cole-Palmer MP-200D-120 Stuart Digital Melting Point Apparatus; 120 VAC (Cole-Palmer Cambridgeshire, UK). 

### 4.1. Compound ***14***

2,4-Difluoronitrobenzene (208 mg, 1.3 mmol) and piperazine (56 mg, 0.65 mmol) were mixed with Et_3_N (132 mg, 1.3 mmol) in EtOH (10 mL) in a Teflon-lined Parr acid digestion bomb. The reactants were heated at 150 °C for 12 h. The vessel was left to cool, and then excess butylamine (191 mg, 2.6 mmol) was added. The reaction vessel was then heated at 150 °C for 12 h again. Once cooled the reaction mixture was diluted with DCM (100 mL) and extracted with water (150 mL) in a separating funnel. The DCM layer was collected then extracted with water (75 mL), dried with MgSO_4_, then filtered. The product was purified by chromatography on flash silica. Elution with DCM/light petroleum ether (25:75) gave the title compound (62 mg, 13%) as yellow crystals, mp 204–205 °C (from dichloromethane:light petroleum ether). λ_max_ (EtOH)/nm 394 (log ε 4.2); ῦ_max_ (diamond)(cm^–1^) 3356s, 2932w, 2864w, 1596s, 1259s, 1240s, 1200s, 1104s, 978s, 740s, 523s and 454s; δ_H_ (400 MHz; CDCl_3_) 1.01 (6H, t, *J* = 8.0), 1.48 (4H, m), 1.67 (4H, m), 3.20 (4H, m), 3.29 (8H, s), 4.81 (2H, NH), 6.16 (2H, s), 6.20 (2H, d, *J* = 8.0) and 8.08 (2H, d, *J* = 8.0); δ_C_ (100.1 MHz; CDCl_3_) 13.7, 20.3, 31.0, 43.0, 51.9, 100.8, 105.7, 130.2, 131.4, 150.7 and 153.4; *m/z* (Orbitrap ASAP) 471.2726 (M^+^ + H, 100%) C_24_H_34_N_6_O_4_H requires 471.2720.

### 4.2. Compound ***16***

2,4-Difluoronitrobenzene (194 mg, 1.2 mmol); 1,4-bis(aminomethyl)benzene (83 mg, 0.6 mmol); Et_3_N (134 mg, 1.3 mmol) and EtOH (10 mL) were mixed in a Teflon-lined Parr acid digestion bomb. The reactants were heated at 150 °C for 12 h. The vessel was left to cool, and excess BuNH_2_ (194 mg, 2.7 mmol) and EtOH (3.5 mL) were added. The reaction vessel was heated at 150 °C for 12 h again. Once cooled, the reaction mixture was diluted with DCM (100 mL) and extracted with water (150 mL) in a separating funnel. The DCM layer was collected then extracted with water (75 mL), dried with MgSO_4_, then filtered. The product was purified by chromatography on flash silica. Elution with DCM/light petroleum ether (25:75) gave the *title compound* (59 mg, 19%) as crystals, mp 203–204 °C (from dichloromethane: light petroleum ether). λ_max_ (EtOH)/nm 406 (log ε 4.3); ῦ_max_ (diamond)(cm^–1^) 3312s, 2956w, 2928w, 2860w, 1616s, 1577s, 1543s, 1458s, 1398s, 1315s, 1251s, 1190s, 1163s, 1129s, 817s, 749s and 556s; δ_H_ (400 MHz; CDCl_3_) 0.94 (6H, t, *J* = 8.0), 1.37 (4H, m), 1.54 (4H, m), 3.09 (4H, m), 4.39 (2H, m, br), 4.51 (4H, d, *J* = 4.0), 5.62 (2H, s), 5.93 (2H, d, *J* = 8.0), 7.37 (4H, s), 8.06 (2H, d, *J* = 8.0) and 8.93 (2H, m, br); δ_C_ (100.1 MHz; CDCl_3_) 13.8, 20.1, 31.0, 42.9, 46.7, 90.9, 104.8, 123.9, 127.6, 129.4, 137.1, 148.2 and 154.3; *m/z* (Orbitrap ASAP) 521.2877 (M^+^ + H, 100%) C_28_H_36_N_6_O_4_H requires 521.2876.

### 4.3. Compound ***17***

2,4-Difluoronitrobenzene (500 mg, 3.14 mmol) in EtOH (30 mL) was mixed with 1,4-diaminobutane (138 mg, 1.57 mmol) and Et_3_N (318 mg, 3.14 mmol). The reaction was heated at 70 °C, cooled, and filtered. The solid was mixed with BuNH_2_ (459 mg, 6.28 mmol) in EtOH (10 mL) in a Teflon-lined Parr acid digestion bomb and heated at 150 °C for 18 h. After cooling, the polar yellow crystals were harvested (103 mg, 13%), mp 207–208 °C. λ_max_ (EtOH)/nm 225 (log ε 3.3) and 398 (3.2); ῦ_max_ (diamond)(cm^–1^) 3318s, 2927w, 2857w, 1615s, 1577s, 1543s, 1460s, 1401s, 1321s, 1247s, 1166s, 815s, 749s, 599s and 533s; δ_H_ (400 MHz; CDCl_3_); *m/z* (Orbitrap ASAP) 473.2877 (M^+^ + H, 100%) C_24_H_36_N_6_O_4_H requires 473.2876.

### 4.4. Crystal Structures

The crystal structures of compounds **14**, **16** and **17** were established using intensity data collected on a Rigaku CCD diffractometer. The structures were routinely solved by dual-space methods using SHELXT [37], and the structural models were completed and optimized by refinement against |*F*|^2^ with SHELXL-2019 [38]. The N-bound hydrogen atom(s) were located in difference maps for compounds **14** and **16,** and their positions were freely refined; for compound **20**, they were geometrically placed and refined as riding atoms. The C-bound hydrogen atoms were placed in idealized locations (C—H = 0.95–0.99 Å) and were refined as riding atoms. The methyl groups were allowed to rotate, but not to tip, to best fit the electron density. The constraint *U*_iso_(H) = 1.2*U*_eq_(carrier) or 1.5*U*_eq_ (methyl carrier) was applied in all cases. The data quality for compound **17** is poorer, perhaps because of some loss of disordered incorporated solvent during data collection, but the structure has been unambiguously established. Full details of the structures and refinements are available in the deposited cifs and CCDC numbers. 

Crystal data for **14** C_24_H_34_N_6_O_4_, pale yellow rod 0.17 × 0.06 × 0.02 mm, *M*_r_ = 470.56, monoclinic, space group *P*2_1_/*n* (No. 14), *a* = 8.1792 (2) Å, *b* = 11.7954 (5) Å, *c* = 12.5512 (4) Å, β = 94.089 (3)°, *V* = 1207.82 (7) Å^3^, *Z* = 2, *T* = 100 K, Cu Kα radiation, λ = 1.54178 Å, μ = 0.734 mm^–1^, ρ_calc_ = 1.294 g cm^–3^, 11,714 reflections measured (10.3 ≤ 2θ ≤ 149.9°), 2422 unique (*R*_Int_ = 0.035), *R*(*F*) = 0.035 [2062 reflections with *I* > 2σ(*I*)], *wR*(*F*^2^) = 0.095 (all data), Δρ_min,max_ (*e* Å^–3^) = –0.18, +0.28, CCDC deposition number 2293919. 

Crystal data for **16** C_28_H_36_N_6_O_4_, pale yellow prism 0.24 × 0.20 × 0.14 mm, *M*_r_ = 520.63, monoclinic, space group *P*2_1_/*n* (No. 14), *a* = 7.1262 (2) Å, *b* = 14.9575 (6) Å, *c* = 12.5881 (6) Å, β = 99.183 (4)°, *V* = 1324.57 (9) Å^3^, *Z* = 2, *T* = 100 K, Cu Kα radiation, λ = 1.54178 Å, μ = 0.724 mm^–1^, ρ_calc_ = 1.305 g cm^–3^, 13,406 reflections measured (9.3 ≤ 2θ ≤ 153.6°), 2736 unique (*R*_Int_ = 0.048), *R*(*F*) = 0.040 [2473 reflections with *I* > 2σ(*I*)], *wR*(*F*^2^) = 0.107 (all data), Δρ_min,max_ (*e* Å^–3^) = –0.20, +0.28, CCDC deposition number 2293920. 

Crystal data for **17** C_24_H_36_N_6_O_4_, very pale yellow chip 0.07 × 0.03 × 0.02 mm, *M*_r_ = 472.59, triclinic, space group *P*$\bar{1}\;$(No. 2), *a* = 10.3217 (10) Å, *b* = 14.5785 (12) Å, *c* = 15.4065 (14) Å, α = 116.407 (9)°, β = 105.981 (8)°, γ = 92.447 (7)°, *V* = 1959.1 (3) Å^3^, *Z* = 3, *T* = 293 K, synchrotron radiation, λ = 0.6889 Å, μ = 0.079 mm^–1^, ρ_calc_ = 1.202 g cm^–3^, 26,619 reflections measured (3.0 ≤ 2θ ≤ 47.0°), 6236 unique (*R*_Int_ = 0.181), *R*(*F*) = 0.144 [2989 reflections with *I* > 2σ(*I*)], *wR*(*F*^2^) = 0.389 (all data), Δρ_min,max_ (*e* Å^–3^) = –0.39, +0.66, CCDC deposition number 2293921. 

## 5. Conclusions

These studies suggest that flexible building blocks are useful for crystallising porous organic zeolites. They appear to allow for additional non-covalent forces, such as cooperative N—H···O and C—H···O hydrogen bonds, to stabilize the extended structure. Two flexible isomers (compounds **1** and **17**) are compared for their mode of packing [1]. Although both structures have a degree of porosity, the first isomer **1** forms channels, and the second more polar isomer **17** forms isolated cavities. The packing is different in each case. In compound **1** the channels are formed from the packing of *n*-butyl chains, but in isomer **17,** the cavities form from six hydrogen-bonded building blocks. The structures are clearly very different and difficult to predict, although butyl chains might be expected to pack together by van der Waals forces, and the ring of hydrogen bonding is logical with hindsight. Holding the functionalised aromatic rings further apart with less conformational freedom, as in compounds **14** and **16**, appears to restrict opportunities for different types of bonding, leading to a porous framework. 

## Figures and Tables

**Table 1:** Key structural properties of **1**, **14**, **16** and **17**.

Compound	Formula	Space Group	H-Bonding Pattern *	Topology
**1**	C_24_H_36_N_6_O_4_	$R\bar{3}$	B	Aligned six-ring pores
**14**	C_24_H_34_N_6_O_4_	*P*2_1/*n*_	C	Dense layered network
**16**	C_28_H_36_N_6_O_4_	*P*2_1/*n*_	D	Dense layered network
**17**	C_24_H_36_N_6_O_4_	$P\bar{1}$	C	Offset six-ring pores

* B = bifurcated N—H···(O,O), D = double acceptor (N—H,C—H)···O, C = cooperative N—H···O + C—H···O′.

## Data Availability

Aberdeen University Library https://www.abdn.ac.uk/library/ (accessed on 23 September 2023).

## Supplementary Materials

The following supporting information can be downloaded at: https://www.mdpi.com/article/10.3390/ijms241914683/s1.
