# Supplementary material for: Porous and Close Packed Supramolecular Assemblies from 2,4-Difluoronitrobenzene with Three Different Linkers and an n-Butylamine Cap
Source: Int J Mol Sci. 2023 Sep 28;24(19):14683. doi: 10.3390/ijms241914683 (PMC10572935; doi:10.3390/ijms241914683)
Supplement: Supplementary file 1 [file ijms-24-14683-s001.zip › ijms-2634960-supplementary.pdf]

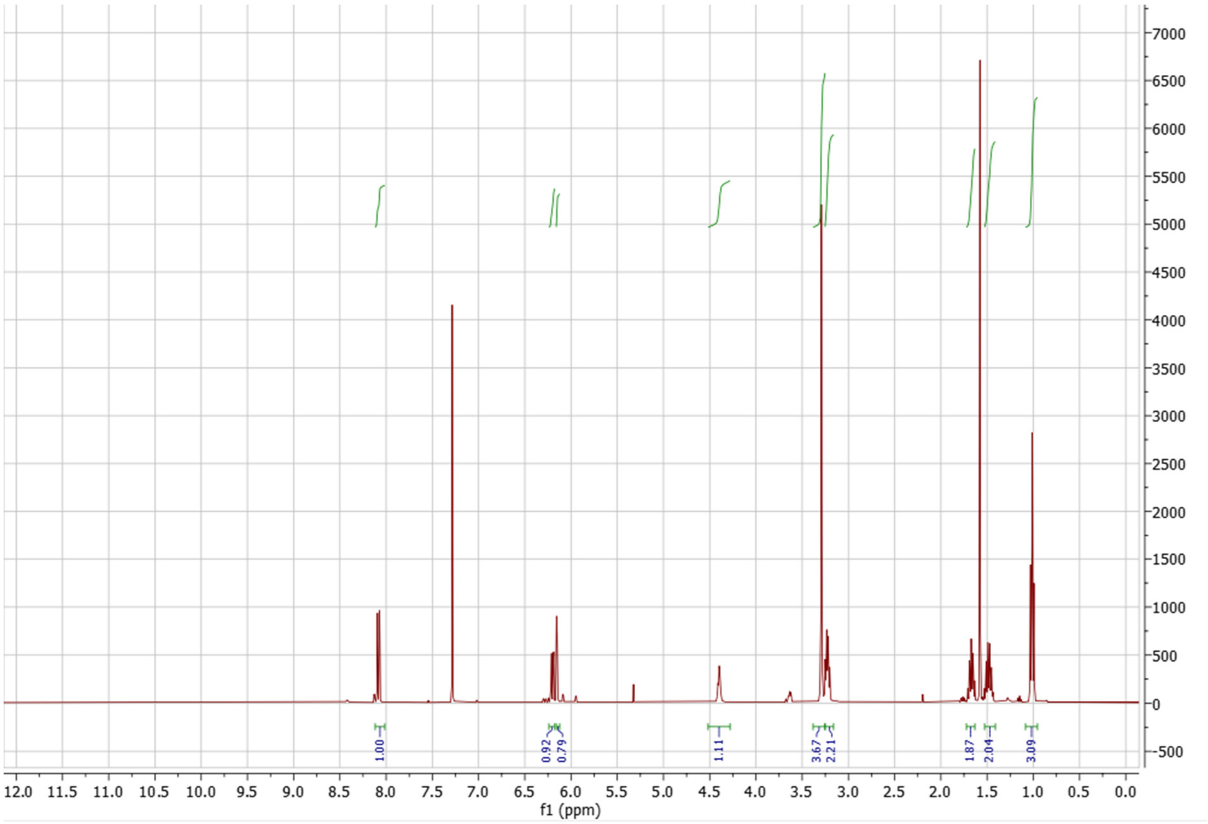

Figure S1. Compound **14** 400 MHz Proton NMR ($CDCl_3$) Full Spectrum 0-12 ppm

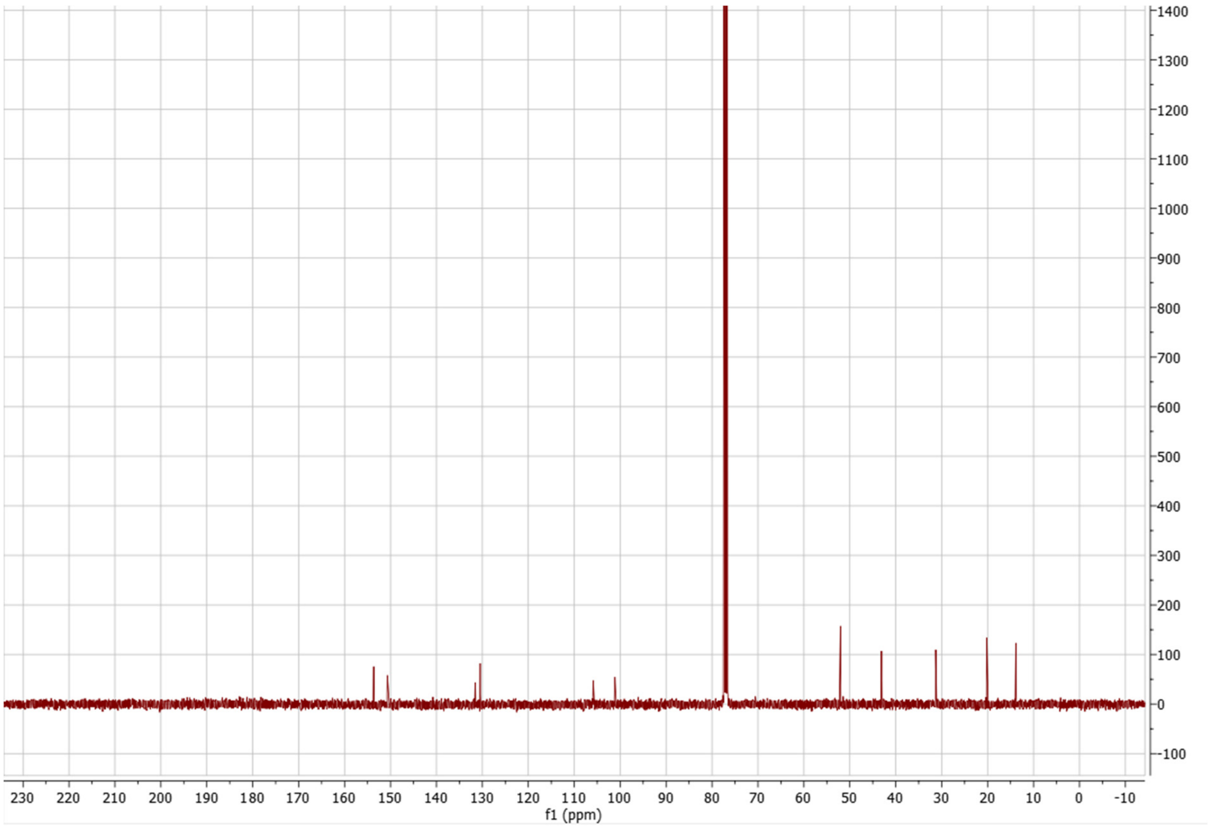

Figure S2. Compound **14** 400 MHz Carbon 13 NMR ($CDCl_3$)

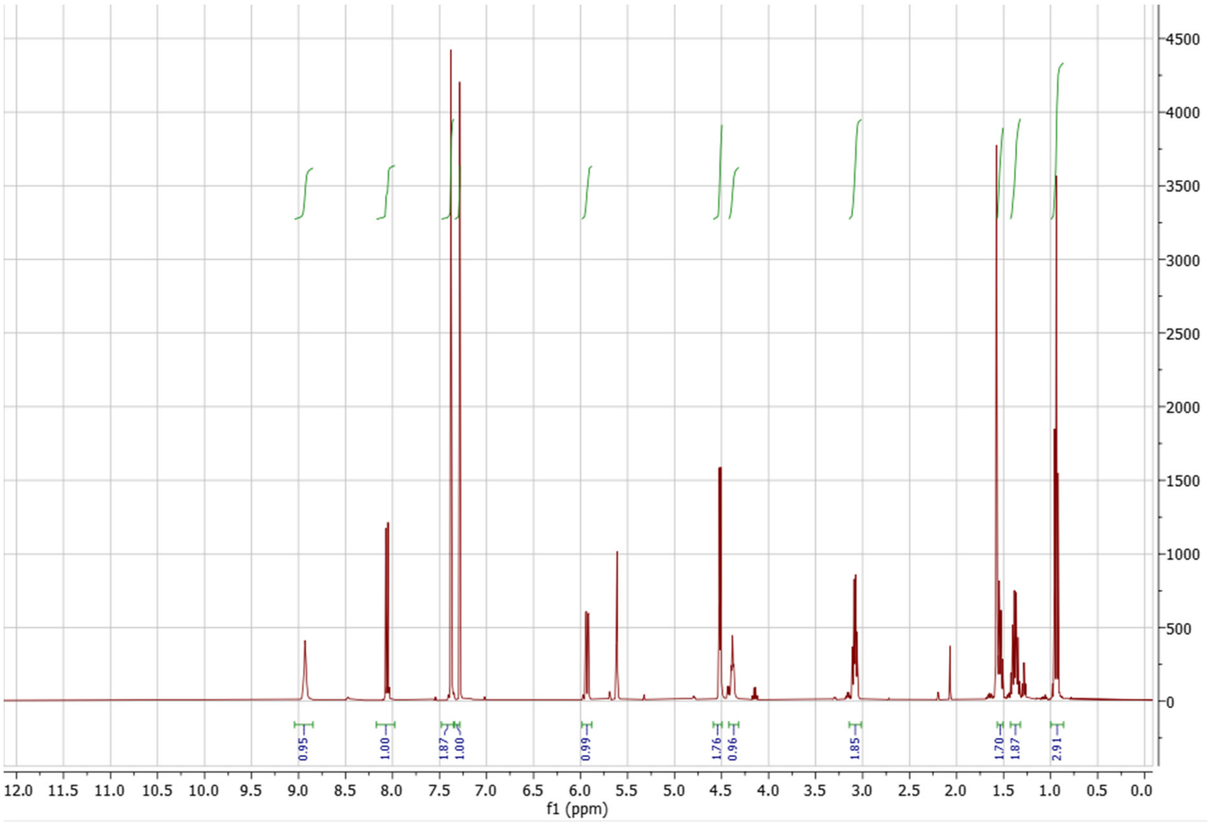

Figure S3. Compound **16** 400 MHz Proton NMR ($CDCl_3$) Full Spectrum 0-12 ppm

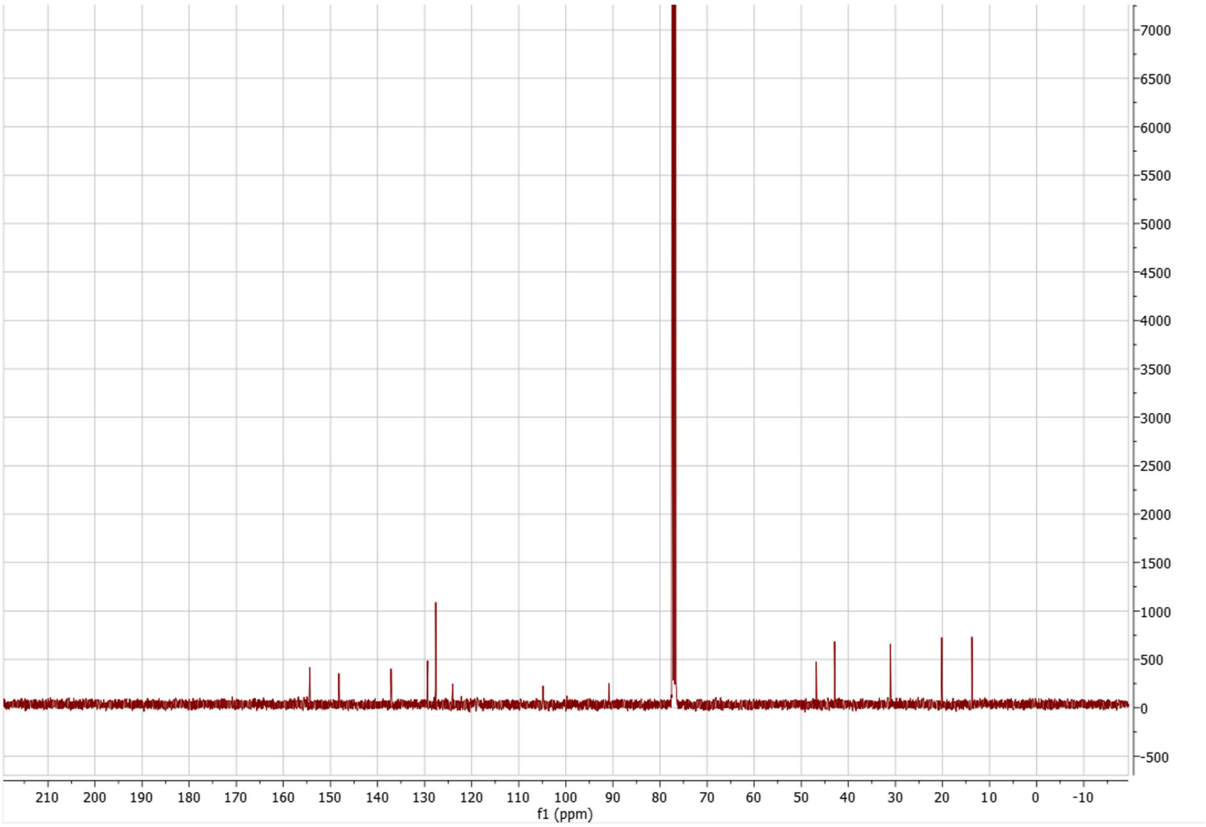

Figure S4. Compound **16** 400 MHz Carbon 13 NMR ($CDCl_3$)
